# Endoscopic management of an ileal Dieulafoy's lesion: a rare cause of severe lower gastrointestinal hemorrhage

**DOI:** 10.1055/a-2971-4045

**Published:** 2026-10-21

**Authors:** Stefania-Raluca Jurcan, Paul Doumbe-Mandengue, Romain Coriat, Flavius-Stefan Marin

**Affiliations:** 1 Gastroenterology and Digestive Oncology Department 26935 Cochin HospitalParisFrance; 2 Paris Cité UniversityParisFrance; 3 Carol Davila University of Medicine and PharmacyBucharestRomania

Ileal Dieulafoy’s lesions are an exceptionally rare cause of life-threatening gastrointestinal hemorrhage. Diagnosis is challenging because of their distal location, the intermittent nature of the bleeding, and the limitations of conventional endoscopy.

A 75-year-old woman with chronic kidney disease and chronic obstructive pulmonary disease was admitted to the intensive care unit with hematochezia, hemodynamic instability, asthenia, and an episode of presyncope. Her hemoglobin level was 7 g/dL, requiring transfusion of 5 U of packed red blood cells.

Contrast-enhanced abdominal computed tomography showed no active bleeding but revealed segmental wall thickening of the ascending colon, raising suspicion of a colonic neoplasm. Upper gastrointestinal endoscopy was normal. Colonoscopy showed no colonic lesions; however, stigmata of recent small-bowel bleeding were noted. Retrograde ileoscopy identified a Dieulafoy’s lesion, 20 cm proximal to the ileocecal valve, initially covered by an adherent clot ( Fig. 1 , Video 1 ). Irrigation dislodged the clot, revealing active bleeding ( Fig. 2 ). Hemostasis was achieved by the submucosal injection of 3 mL of diluted epinephrine (1:10,000), followed by the placement of three 9-mm hemostatic clips ( Fig. 3 ). No further bleeding was observed. The patient was discharged 2 days later. The hemoglobin level remained stable from discharge to the 1-month follow-up (10 g/dL), with no recurrent bleeding.

**Fig. 1:**
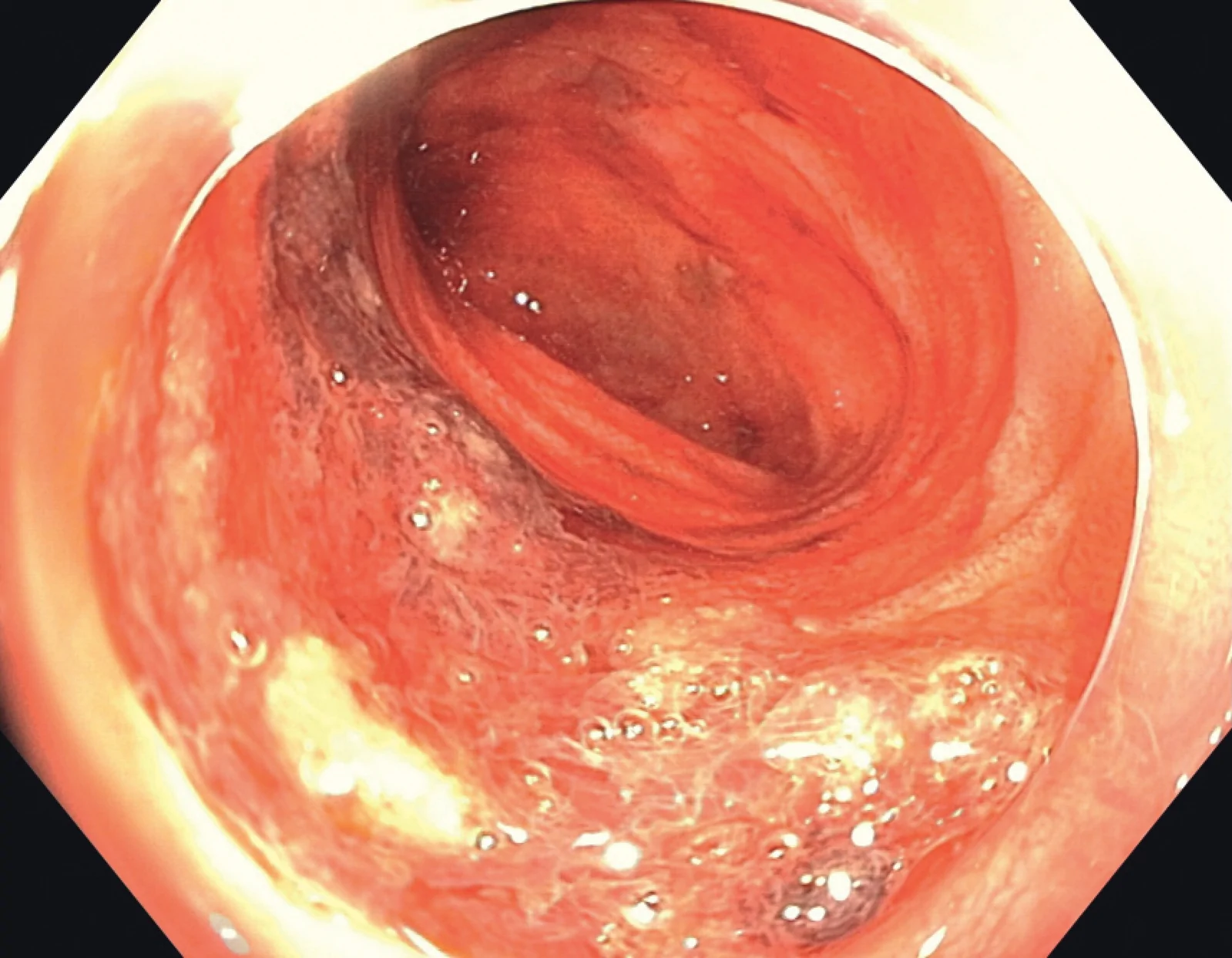
An adherent clot covering an ileal Dieulafoy’s lesion.

**Video 1** Endoscopic management of an ileal Dieulafoy’s lesion causing hemorrhagic shock. URI:


**Fig. 2:**
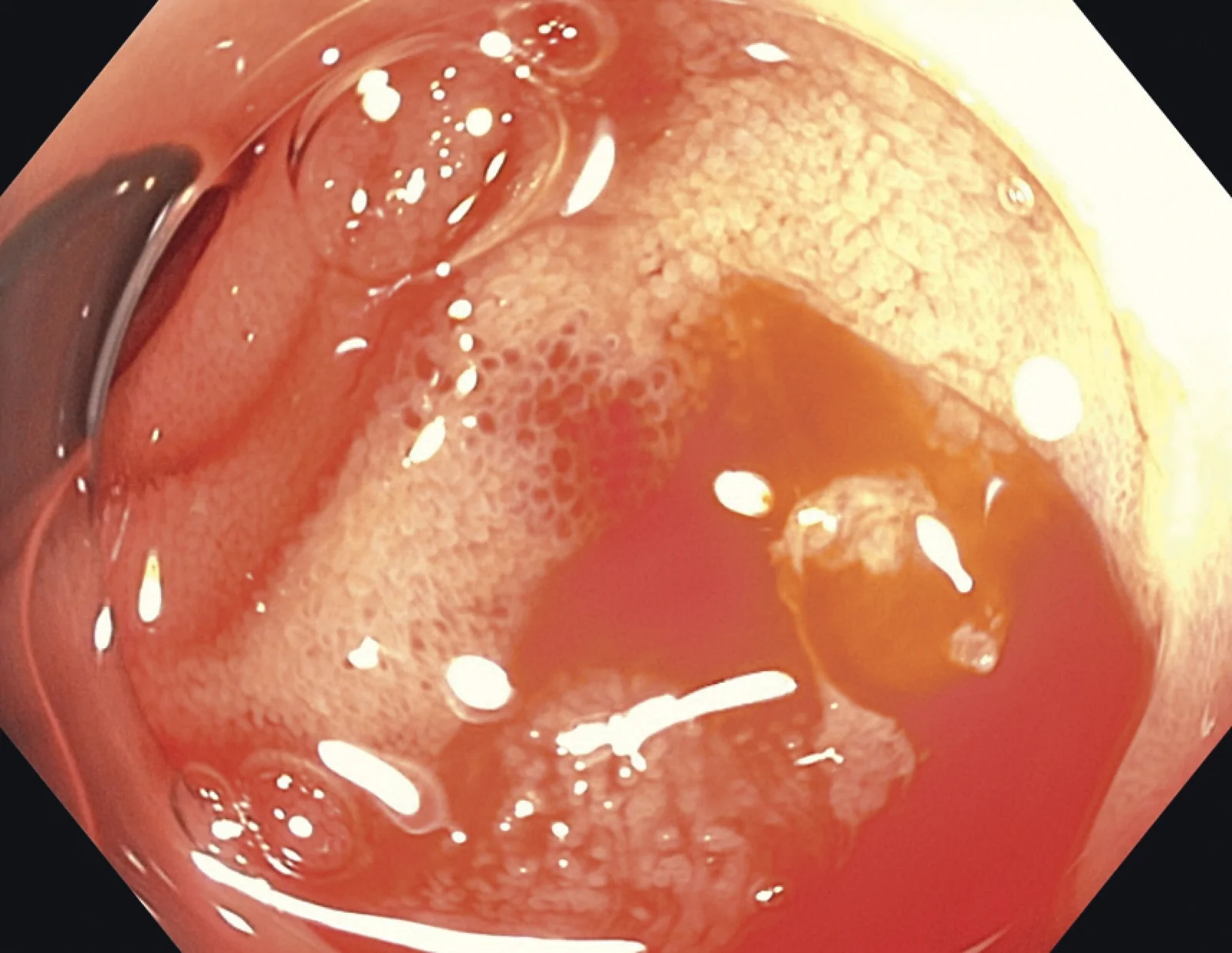
Active bleeding after removal of the adherent clot.

**Fig. 3:**
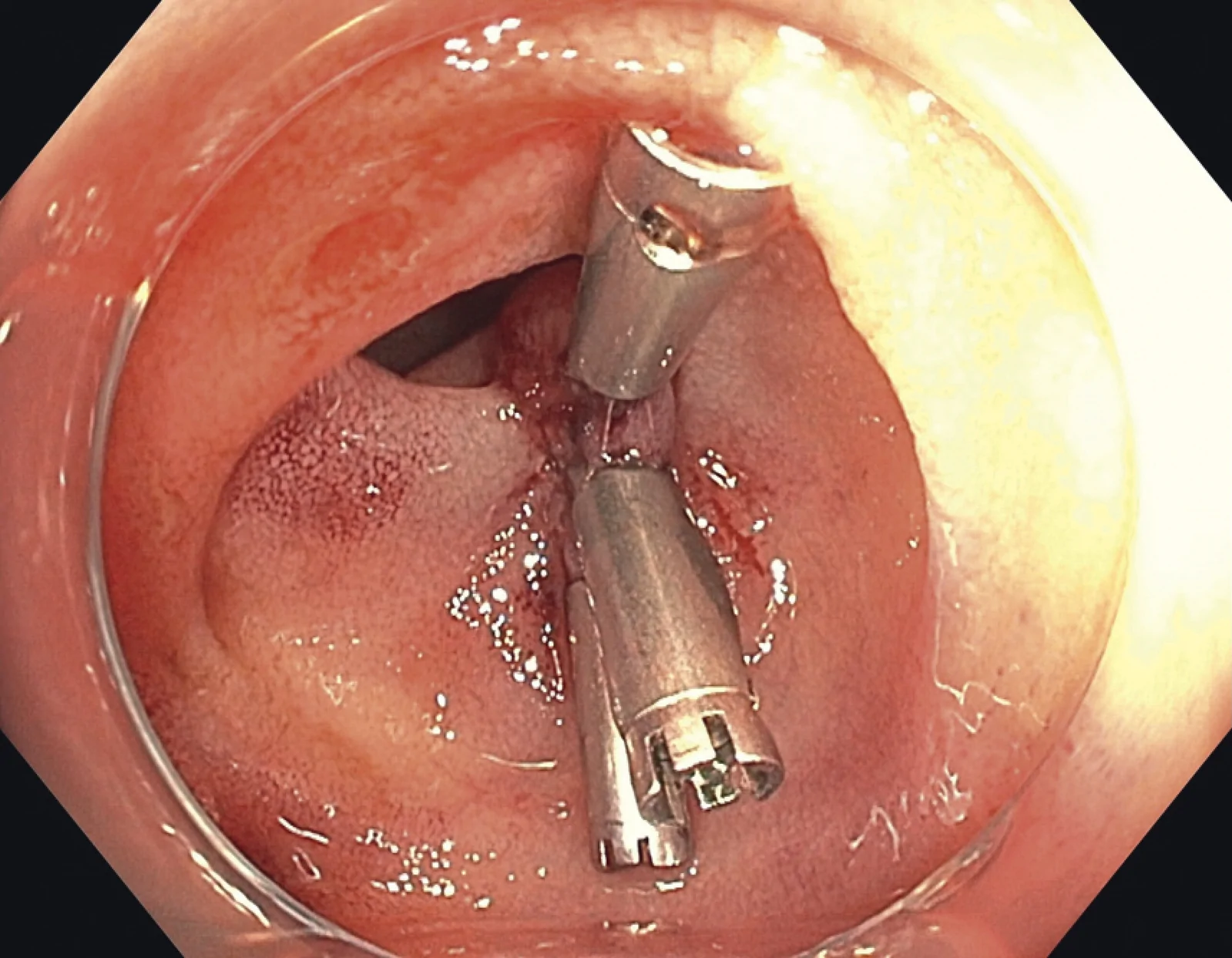
Hemostasis achieved after epinephrine submucosal injection and placing of hemostatic clips.

The ileal Dieulafoy’s lesion remains an uncommon cause of severe hemorrhage. Among small-bowel Dieulafoy’s lesions, the ileum is the least frequently involved site, reported far less often than the jejunum or duodenum. 1​ Reported ileal cases have mainly been managed by device-assisted enteroscopy or surgical resection. 1​ ​2​ Hemostasis achieved during colonoscopy with retrograde ileoscopy, as in the present case, is seldom described. Although its risk factors resemble those of angiodysplasia (advanced age, chronic kidney disease, chronic obstructive pulmonary disease, and cardiovascular disease), 3​ its rebleeding rate may be higher. 4​ Endoscopic therapy is safe and effective, with surgery reserved for failures.

Endoscopy_UCTN_Code_TTT_1AQ_2AZ

## Declaration of GenAI Use

The authors confirm that no generative artificial intelligence was used in the preparation of this manuscript.
